# Photocatalytic Oxidative Bromination of 2,6-Dichlorotoluene to 2,6-Dichlorobenzyl Bromide in a Microchannel Reactor

**DOI:** 10.1021/acsomega.1c06737

**Published:** 2022-01-26

**Authors:** Lin Liu, Peng Liu, Di Zhang, Hong-Yu Zhang, Yuecheng Zhang, Jiquan Zhao

**Affiliations:** † School of Chemical Engineering, Hebei University of Technology, Tianjin 300401, P.R. China; ‡ Tasly Pharmaceutical Group Co. Ltd., Tianjin 300402, P.R. China; § Hebei Provincial Key Lab of Green Chemical Technology and High Efficient Energy Saving, School of Chemical Engineering, Hebei University of Technology, Tianjin 300401, P.R. China

## Abstract

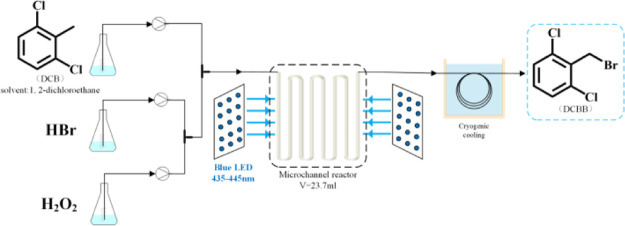

Photocatalytic oxidative benzylic bromination with hydrobromic acid (HBr) and hydrogen peroxide (H_2_O_2_) is a green process for the synthesis of benzyl bromides, but suffers from the risk of explosion when performing it in a batch reactor. This disadvantage could be overcome by running the reaction in a microchannel reactor. In this work, a green and safe process for the synthesis of 2,6-dichlorobenzyl bromide (DCBB) was developed by conducting selective benzylic bromination of 2,6-dichlorotoluene (DCT) with H_2_O_2_ as an oxidant and HBr as a bromine source in a microchannel reactor under light irradiation. The reaction parameters were optimized, and the conversion of DCT reached up to 98.1% with a DCBB yield of 91.4% under the optimal reaction conditions.

## 1 Introduction

**Figure 1:**
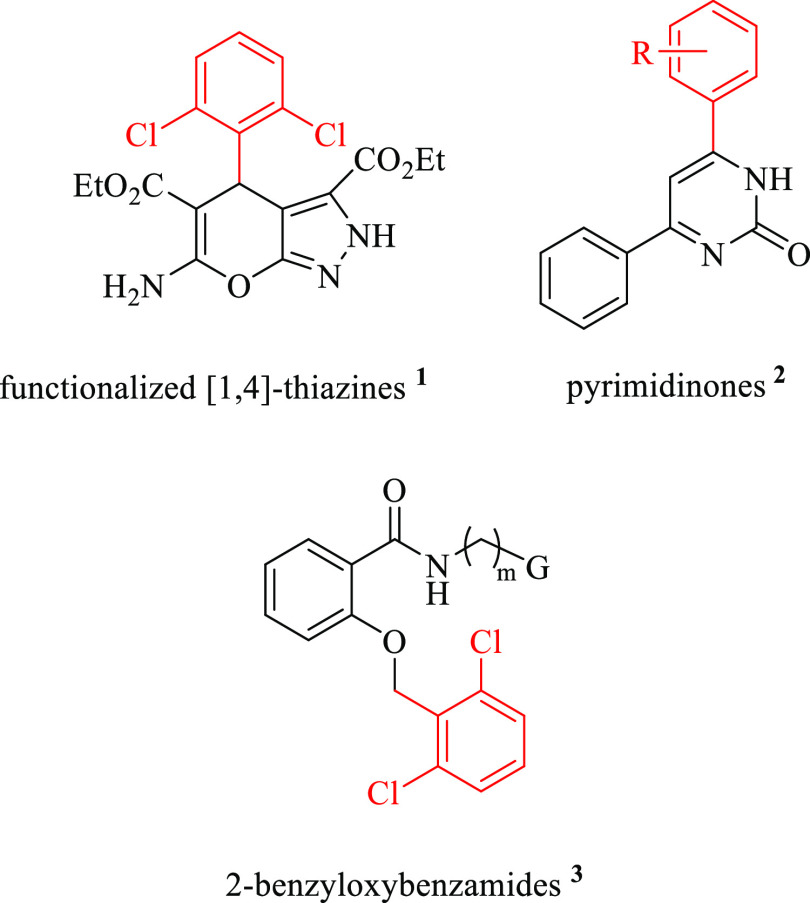
Bioactive compounds from DCBB.

2,6-Dichlorobenzyl bromide (DCBB) is an important intermediate in the synthesis of bioactive molecules such as functionalized [1,4]-thiazines, 4,6-diarylpyrimidin-2(1*H*)-ones, and 2-benzyloxybenzamides (Figure 1).^1−3^ DCBB is commonly obtained by benzylic bromination of 2,6-dichlorotoluene (DCT) with bromine in the presence of the free radical initiator or under light irradiation.^4,5^ The benzylic bromination with bromine suffers from the disadvantages of the low utilization rate of bromine due to transformation of half bromine to hydrogen bromide as a byproduct and dangers in transport and storage of bromine due to its toxicity and high vapor pressure.^6^ Therefore, many reagents and protocols instead of bromine have been developed for the selectively benzylic bromination, such as H_2_O_2_/HBr/NBS,^7^ BBr_3_,^8^ and NBS/SiCl_4_,^9^ and various oxidative bromination systems including NaBrO_3_/NaHSO_3_,^10^ NaBrO_3_/KBr^–^/H^+^,^11^ KBr/Oxone,^12^ NaNO_2_/KBr/HCl,^13^ and HBr/H_2_O_2_.^14−17^ Among them, the oxidative bromination with HBr/H_2_O_2_ is the most recommended because of low cost of both hydrobromic acid (HBr) and H_2_O_2_, 100% utilization of bromine source, and water as the only byproduct avoiding the environmental problems being frequently involved with other oxidants.^18^

Traditionally, the oxidative bromination was performed in a batch reactor,^14,17^ which suffers from the disadvantages of low reaction efficiency owing to the short radiation distance of light, risk of explosion, especially in large-scale production.

In recent years, significant progresses have been achieved in microchannel reactor technology for the chemical transformations with the advancement of science and technology.^19−22^ Compared with traditional batch reactors, microchannel reactors have the essential characteristics of high efficiencies of mass and heat transfer, as well as an enhanced specific surface area, affording its high safety, good operability, precise control of the reaction conditions for getting high selectivity of the target product, and easiness of scale-up. Actually, microchannel reactors have been found to have wide applications in the field of photocatalytic chemistry for their abovementioned advantages and easy reaching reactants of light, including photoredox catalysis,^23^ elemental fluorination of β-dicarbonyl compounds,^24^ conjugate addition of acrolein with glycosylradicals,^25^ trifluoromethylation of heterocycles,^26^ oxidation of benzene to phenol,^27^ and light-initiated benzylic chlorination^28^ as well as alicyclic compounds.^29,30^ In particular, microreactors^31−34^ were also applied to the benzylic bromination with Br_2_ or HBr/H_2_O_2_ under light irradiation. On the other hand, it is expected that the oxidative bromination with HBr/H_2_O_2_ in a microchannel reactor can reduce the severe decomposition of H_2_O_2_ catalyzed by bromine generated in situ in a batch reactor,^35^ due to the less contact time of reactants in the microchannel reactors.

Herein, the benzylic bromination of DCT with HBr as the bromine source and H_2_O_2_ as the oxidant was conducted in a microchannel reactor under light irradiation for the safe and environmentally friendly production of DCBB. The effects of reaction temperature, reactant molar ratios, residence time, and light intensity as well as material concentration were investigated, from which the optimal reaction conditions for the preparation of DCBB from DCT were obtained.

## 2 Results and Discussion

### 2.1 Effect of Temperature

**Figure 2:**
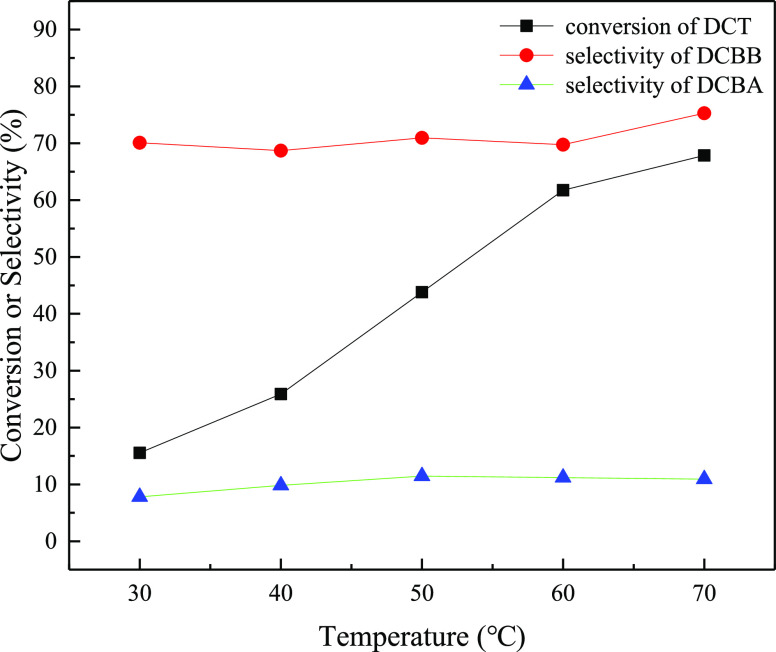
Effect of temperature on the oxidative benzylic bromination of DCT.

For the benzylic oxidative bromination of DCT in a microchannel reactor irradiated with a light of specific wavelength and intensity, the reaction temperature is a key factor to affect the reaction. Therefore, the effect of reaction temperature on the reaction was investigated first. The main reaction products were determined to be DCBB and DCBA. No aryl substitution products were observed, indicating excellent selectivity toward benzylic substitution of the reaction in the microchannel reactor under the reaction conditions. As shown in Figure 2, the conversion of DCT increased from 15.5 to 67.8% with the temperature increased from 30 to 70 °C, and the selectivity of DCBB increased from 68.7 to 75.3%. Both DCT conversion and DCBB selectivity increased slightly with a further increase in reaction temperature above 70 °C. Meanwhile, the selectivity of DCBA almost remained constant around 10.2% in all the cases.

Reaction conditions: 87 W blue light; HBr/H_2_O_2_:DCT (molar ratio) = 1.3:1.3:1; solution A: 15.0 wt % solution of DCT (0.093 mol) in 1,2-dichloroethane (73.0 mL), 1.47 mL/min; solution B: 12.5 wt % of HBr aqueous solution, 1.3 mL/min; solution C: 5.76 wt % of H_2_O_2_ aqueous solution, 1.3 mL/min; residence time = 5.82 min; reaction pressure = 0.8 MPa.

### 2.2 Effect of the Amount of HBr and H_2_O_2_

**Figure 3:**
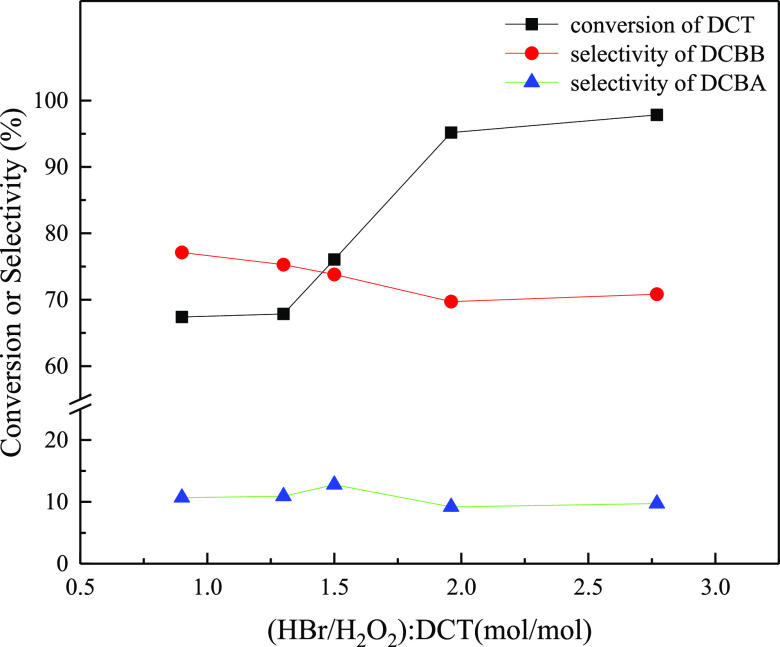
Effect of (HBr/H_2_O_2_): DCT (molar ratio) on the oxidative benzylic bromination of DCT.

Next, the effect of the molar ratios of HBr/H_2_O_2_:DCT on the benzylic oxidative bromination was studied. As shown in Figure 3, the conversion of DCT increased with HBr/H_2_O_2_/DCT molar ratios and reached 95.2% at 1.96:1.96:1, but the selectivity of DCBB decreased with the increase in HBr/H_2_O_2_/DCT molar ratios. Both the conversion of DCT and the selectivity of DCBB changed slightly with a further increase in HBr/H_2_O_2_/DCT molar ratios. The selectivity of DCBA changed slightly around 10.4% with the variation of HBr/H_2_O_2_/DCT molar ratios. To gain DCBB in high yield with low consumption of HBr and H_2_O_2_, the optimal HBr/H_2_O_2_/DCT molar ratios were determined to be 1.5:1.5:1. In this case, the conversion of DCT was 76.1% with a DCBB selectivity of 73.8%. The results indicated the superiority of an oxidative bromination with H_2_O_2_/HBr in a microchannel reactor compared to that in a batch reactor in view of low H_2_O_2_ consumption. The optimal H_2_O_2_ to HBr molar ratio was around 2:1 in a similar oxidative bromination in a batch reactor.^35^

Reaction conditions: 87 W blue light; solution A: 15.0 wt % solution of DCT (0.093 mol) in 1,2-dichloroethane (73.0 mL); solution B: 12.5 wt % of HBr aqueous solution; solution C: 5.76 wt % of H_2_O_2_ aqueous solution; residence time = 5.83 min; reaction pressure = 0.8 MPa; reaction temperature = 70 °C.

### 2.3 Effect of Residence Time

**Figure 4:**
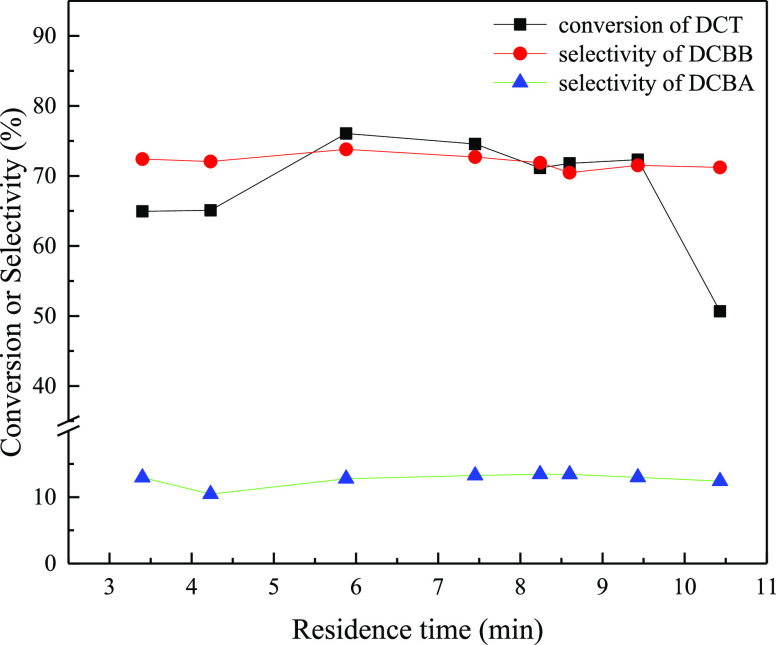
Effect of residence time on the oxidative benzylic bromination reaction of DCT.

In a microchannel reactor, the residence time of reactants is another key factor to affect the reaction, and it also indirectly reflects the irradiation time of light on the reactants in a photocatalytic reaction. Therefore, the effect of residence time on the reaction was investigated at 70 °C and HBr/H_2_O_2_/DCT molar ratios of 1.5:1.5:1. As shown in Figure 4, the conversion of DCT increased with the increase in residence time initially, reached its maximum of 76.1% at the residence time of 5.88 min, then decreased slowly with the residence time until the residence time reached 9.43 min, and then dropped sharply. The sharp decline of DCT above the residence time of 9.43 min could be ascribed to the poor mixing of aqueous and organic phases in the microchannel reactor at long residence time. The selectivity of DCBB increased slowly with residence time and also reached its maximum of 73.8% at the residence time of 5.88 min and then always decreased slightly with further extending residence time. Similar to the other cases, the selectivity of DCBA changed slightly around 12.7% with residence time. Thus, the residence time was determined to be 5.88 min.

Reaction conditions: 87 W blue light; HBr/H_2_O_2_/DCT (molar ratio) = 1.5:1.5:1; solution A: 15.0 wt % solution of DCT (0.093 mol) in 1,2-dichloroethane (73.0 mL); solution B: 12.5 wt % of HBr aqueous solution; solution C: 5.76 wt % of H_2_O_2_ aqueous solution; reaction pressure = 0.8 MPa; reaction temperature = 70 °C.

### 2.4 Effect of Light Intensity

As can be seen from Table 1, the DCT was essentially unreactive in dark, and no DCBB was detected (Table 1, entry 1). With the light on, the reaction took place. As the light intensity increased from 72 to 87 W, the DCT conversion increased from 66.7 to 76.1% with the decrease of DCBB selectivity from 76.2 to 73.8% but with the increase of DCBA selectivity from 9.5 to 12.8% (Table 1, entries 2 and 3). With a further increase in light intensity, the DCT conversion increased slowly, but the selectivities of both DCBB and DCBA decreased (Table 1, entry 4), ascribing to other side reactions occurred under excessive light intensity. Therefore, the light intensity was determined to be 87 W.

**Table 1:** Effect of Light Intensity on the Bromination of DCT with HBr–H_2_O_2_

			product selectivity/%	
entry	conditions	DCT conversion/%	DCBB	DCBA
1	dark			
2	72 W blue light	66.7	76.2	9.5
3	87 W blue light	76.1	73.8	12.8
4	118 W blue light	78.4	72.5	7.6

Reaction conditions: HBr/H_2_O_2_/DCT (molar ratio) = 1.5:1.5:1; solution A: 15.0 wt % solution of DCT (0.093 mol) in 1,2-dichloroethane (73.0 mL), 1.37 mL/min; solution B: 12.5 wt % of HBr aqueous solution, 1.33 mL/min; solution C: 5.76 wt % of H_2_O_2_ aqueous solution, 1.33 mL/min; residence time = 5.88 min; reaction pressure = 0.8 MPa; reaction temperature = 70 °C.

### 2.5 Effect of Reactant Concentrations

**Figure 5:**
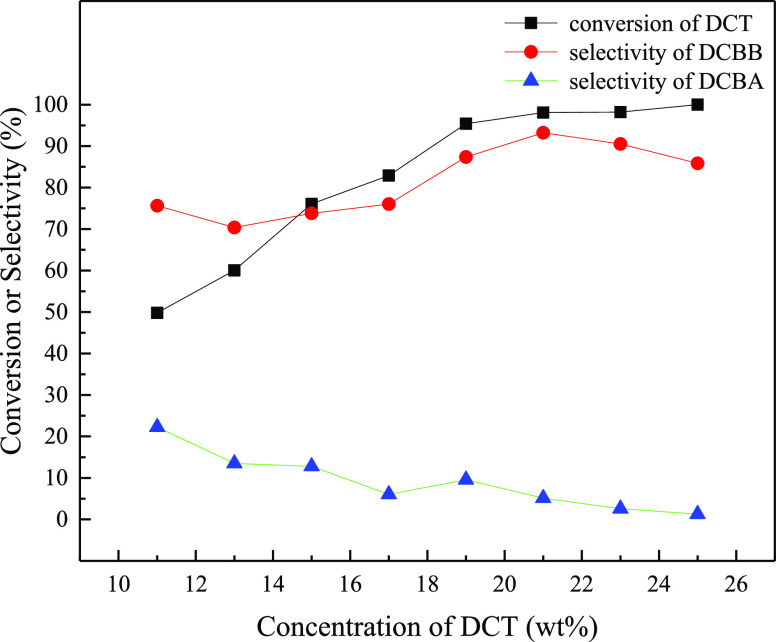
Effect of solution concentration on the oxidative benzylic bromination reaction of DCT.

Under the molar ratios of HBr/H_2_O_2_/DCT = 1.5:1.5:1, a residence time of 5.88 min, and a light of intensity of 87 W, the effect of DCT concentration on the reaction was investigated. As shown in Figure 5, the conversion of DCT increased from 49.8 to 98.1% with the increase of DCT concentration from 11.0 to 21.0 wt % and then changed slowly with a further increase in DCT concentration. In general, the selectivity of DCBB presented the trend that first increased, then decreased with the increase in DCT concentration, and reached its maximum of 93.2% at the DCT concentration of 21.0 wt %. In addition, the selectivity of DCBA displayed a gradual descending trend with the increase in DCT concentration. The maximum DCBB yield of 91.4% was obtained when the DCT concentration was 21.0 wt %.

Reaction conditions: 87 W blue light; HBr/H_2_O_2_/DCT (molar ratio) = 1.5:1.5:1; solution A: the solution of DCT in 1,2-dichloroethane, 1.37 mL/min; solution B: HBr aqueous solution, 1.33 mL/min; solution C: H_2_O_2_ aqueous solution, 1.33 mL/min; residence time = 5.88 min; reaction pressure = 0.8 MPa; reaction temperature = 70 °C.

### 2.6 Effect of H_2_O_2_ to HBr Molar Ratio on the Reaction

**Figure 6:**
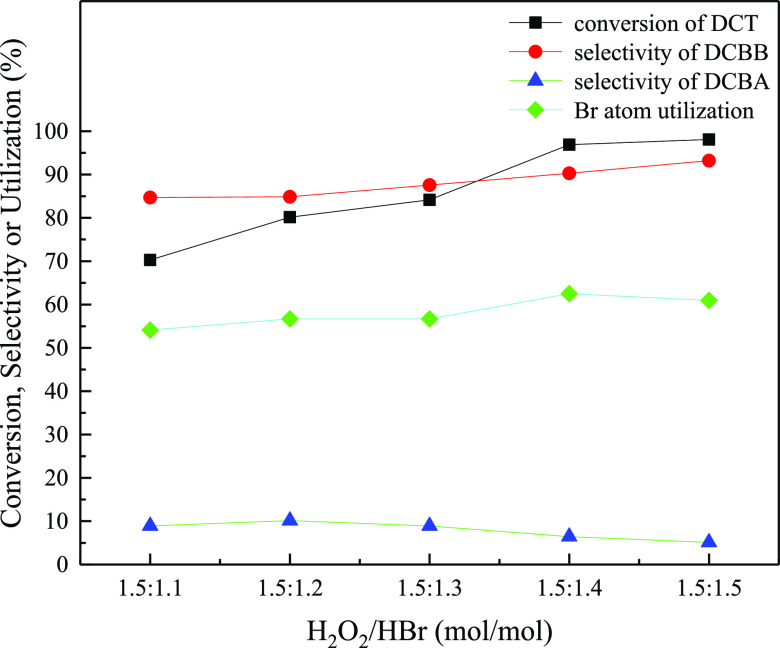
Effect of H_2_O_2_/HBr (molar ratio) on the oxidative benzylic bromination of DCT.

It is of importance to optimize the molar ratio of H_2_O_2_/HBr to improve utilization efficiency of H_2_O_2_ or Br. The optimization was performed under the respective DCT, HBr, and H_2_O_2_ concentrations of 21.0, 16.3, and 7.7 wt % and the DCT to H_2_O_2_ of 1:1.5. As shown in Figure 6, both the conversion of DCT and the selectivity of DCBB decreased slightly with the increase in the H_2_O_2_ to HBr molar ratio from 1.5:1.5 to 1.5:1.4 and showed a downward trend with a further increase in the H_2_O_2_ to HBr molar ratio. However, the maximum Br utilization efficiency of 62.5% was obtained at the H_2_O_2_ to HBr molar ratio of 1.5:1.4. Therefore, the optimal molar ratio of H_2_O_2_ to HBr was 1.5:1.4.

Reaction Conditions: 87 W blue light; DCT/H_2_O_2_ (molar ratio) = 1:1.5; solution A: 21.0 wt % solution of DCT (0.141 mol) in 1,2-dichloroethane (73.0 mL); solution B: 16.3 wt % of HBr aqueous solution; solution C: 7.1 wt % of H_2_O_2_ aqueous solution; residence time = 5.88 min; reaction pressure = 0.8 MPa; reaction temperature = 70 °C.

Based on the abovementioned experimental results, the optimal parameters for the oxidative benzylic bromination of DCT to DCBB in a microchannel reactor under light irradiation were obtained, which are HBr/H_2_O_2_/DCT molar ratios of 1.5:1.5:1, DCT, HBr, and H_2_O_2_ concentrations of 21.0, 16.3, and 7.7 wt %, residence time of 5.88 min, reaction pressure of 0.8 MPa, reaction temperature of 70 °C, and irradiation with 87 W blue light. Under the optimal conditions, the conversion of DCT was 98.1% with a DCBB selectivity of 93.2%, corresponding to the DCBB yield of 91.4%.

## 3 Conclusions

A safe and green process for the synthesis of DCBB from oxidative benzylic bromination of DCT has been developed by performing the reaction in a microchannel reactor with H_2_O_2_ as the oxidant and HBr as the bromine source under light irradiation. Under optimal reactions, the conversion of DCT reached up to 98.1% with a DCBB yield of 91.4%. The high efficiency of the reaction could be ascribed to the strong operability and easy access of light to reactants of the microchannel reactor.

## 4 Experimental Section

### 4.1 Reagents and Instruments

All reagents were of analytical grade and used as received without further purification. DCT, 1,2-dichloroethane, aqueous hydrogen peroxide (H_2_O_2_, 30.0 wt %), and HBr (47.0 wt %) were provided by Shanghai Titan Chemical Reagent Cooperation. Deionized water was prepared by ourselves in our laboratory.

The oxidative benzylic bromination of DCT under light irradiation was carried out in a Kiloflow-type continuous flow microchannel reactor from Chemtrix B.V. (Echt, The Netherlands). The light source is composed of three light plates with a power of 5 W and two light strips with a power of 36 W, affording a blue light with a wavelength of 435–445 nm. The reaction is illuminated by placing the light plates and light bars 5 mm from the glass reaction plate of the microchannel reactor. The reaction mixture was analyzed on a Shimadzu high-performance liquid chromatograph (HPLC) equipped with a Shim-pack VP-ODS C_18_ column.

### 4.2 Oxidative Bromination of DCT

**Figure 7:**
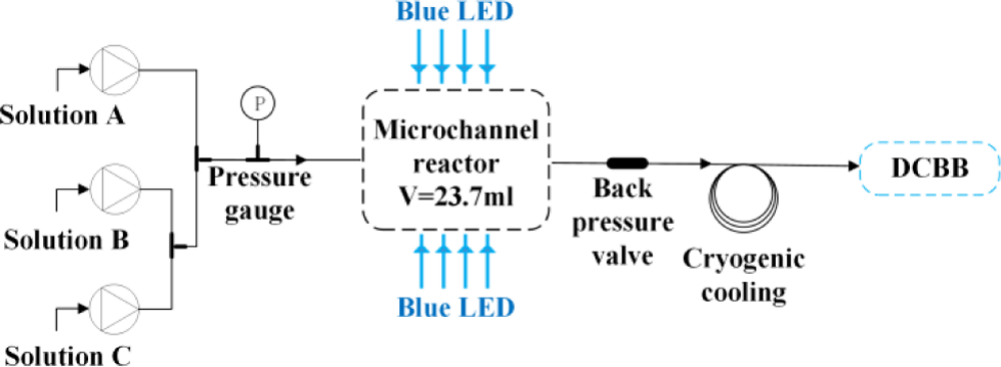
Process flow of oxidative bromination under light irradiation.

In a typical experiment, 22.7 g (0.141 mol) of DCT and 73.0 mL of 1,2-dichloroethane were added into an Erlenmeyer flask successively to give solution **A**. Into another Erlenmeyer flask was added 26.0 g of 30.0 wt % H_2_O_2_, followed by dilution with 75.2 mL of deionized water to afford solution **B**. Then, 40.0 g of 47.0 wt % HBr was introduced into the third Erlenmeyer flask, followed by dilution with 75.1 mL of deionized water to afford solution **C**. As shown in Figure 7, the solutions **B** and **C** were mixed through a three-way valve first and met with solution **A** in the second valve pumped by three 10 mL liquid feed pumps. The mixture finally entered the microchannel reactor to complete the reaction at given temperature and pressure. The reaction liquid flowed out of the reactor and was quenched at low temperature in a cold trap (0 °C). The reactor pressure is monitored by a pressure gauge at the inlet and controlled by a back pressure valve at the outlet throughout the process.

After reaction, the reaction mixture was analyzed by HPLC. First, a standard curve was established using high performance liquid chromatography (HPLC) for the quantitative analysis of DCT, DCBB, and 2,6-dichlorobenzoic acid (DCBA).

The conversion rate of DCT and the selectivities toward DCBB and DCBA were calculated using the following formulas
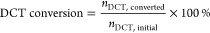
1
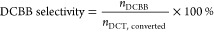
2
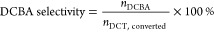
3*n*_DCT, initial_ and *n*_DCT, converted_ are the initial and converted amounts of DCT in moles, respectively. *n*_DCBB_ and *n*_DCBA_ are the amounts of DCBB and DCBA formed in moles, respectively.
